# Improving immunological insights into the ferret model of human viral infectious disease

**DOI:** 10.1111/irv.12687

**Published:** 2019-10-03

**Authors:** Julius Wong, Daniel Layton, Adam K. Wheatley, Stephen J. Kent

**Affiliations:** ^1^ Department of Microbiology and Immunology Peter Doherty Institute for Infection and Immunity University of Melbourne Melbourne Vic. Australia; ^2^ CSIRO Health and Biosecurity Australian Animal Health Laboratories Geelong Vic. Australia; ^3^ Melbourne Sexual Health Centre and Department of Infectious Diseases Alfred Hospital and Central Clinical School Monash University Melbourne Vic. Australia; ^4^ ARC Centre for Excellence in Convergent Bio‐Nano Science and Technology University of Melbourne Parkville Vic. Australia

**Keywords:** ferret, immunology, influenza

## Abstract

Ferrets are a well‐established model for studying both the pathogenesis and transmission of human respiratory viruses and evaluation of antiviral vaccines. Advanced immunological studies would add substantial value to the ferret models of disease but are hindered by the low number of ferret‐reactive reagents available for flow cytometry and immunohistochemistry. Nevertheless, progress has been made to understand immune responses in the ferret model with a limited set of ferret‐specific reagents and assays. This review examines current immunological insights gained from the ferret model across relevant human respiratory diseases, with a focus on influenza viruses. We highlight key knowledge gaps that need to be bridged to advance the utility of ferrets for immunological studies.

## 1 INTRODUCTION

Animal models have proved critical in developing concepts of immunity to infection. Non‐human primates (NHP) are a highly human‐relevant disease model for infectious agents due to high genetic conservation between humans and NHP.^1^, ^2^, ^3^ However, significant cost and ethical issues often necessitate the use of smaller animal models for both basic and translational research. Mice (*Mus musculus*) are a favoured small animal model due to widespread availability, incisive transgenic models, comprehensive genomic information^4^ and readily available reagents. However, for many viruses, alternative animal models better recapitulate human physiology and disease. Ferrets (*Mustela putorius furo*) have been employed to study the pathogenesis of a variety of human pathogens, including human and avian influenzas, coronaviruses including severe acute respiratory syndrome (SARS‐CoV),^5^, ^6^, ^7^, ^8^, ^9^, ^10^, ^11^, ^12^, ^13^, ^14^ human respiratory syncytial virus (HRSV),^15^, ^16^, ^17^, ^18^, ^19^, ^20^ human metapneumovirus (HMPV),^21^ Ebola virus^22^, ^23^, ^24^, ^25^, ^26^, ^27^, ^28^ and henipavirus (Nipah virus and Hendra virus).^29^, ^30^, ^31^, ^32^, ^33^, ^34^ While ferrets can be productively infected with many of these viruses, a lack of some tools to interrogate ferret immunological responses to infection limits insights that might impact the development of vaccines and/or therapeutics. Here, we review recent insights gained from ferret models of human respiratory diseases, with a major focus on influenza, and highlight several knowledge gaps whose closure will greatly enhance the informational gain from ferret models to advance development of human vaccines and therapeutics.

## 2 FERRETS AS AN INFLUENZA PATHOGENESIS AND TRANSMISSION MODEL

Ferrets can be directly infected with influenza from human clinical isolates without prior adaptation. Influenza infection of ferrets recapitulates key hallmarks of human clinical disease, such as high fever accompanied by sweating, as well as respiratory symptoms such as rhinorrhea and sternutation.^35^, ^36^ The shared susceptibility to influenza infection is based on similarity in respiratory tract physiology, where a predominance of α2,6‐linked sialic acid (SA) receptors in the upper respiratory tract of ferrets mimics that of humans,^37^, ^38^, ^39^, ^40^, ^41^ unlike α2,3‐SA prevalent in other species such as mice. Ferrets are an extremely valuable model for studies on influenza pathogenesis^42^, ^43^ and both direct and aerosol transmission.^44^, ^45^ Critically, ferrets are a susceptible host for highly pathogenic avian strains of influenza with pandemic potential, such as H5N1 and H7N9, although disease severity in infected ferrets is somewhat variable.^42^, ^46^, ^47^, ^48^ Similar variability in pathogenesis has been reported for some seasonal strains such as recent H3N2 isolates,^49^, ^50^ which display a range of disease severity in humans^51^ but generally remain mild in ferrets.^52^, ^53^

## 3 FERRETS FOR INFLUENZA SURVEILLANCE AND VACCINE DEVELOPMENT

Ferrets play a critical role in annual seasonal influenza vaccine strain selection. Antigenic drift in circulating strains is monitored primarily using hemagglutination inhibition (HI) assays on serum from ferrets infected with recently circulating human viral isolates.^54^, ^55^ In both ferrets and humans, HI titres are a marker of protection from acquiring infection and currently the key immunological correlate for assessing potential vaccine effectiveness. Currently licensed seasonal vaccines, historically trivalent (TIV) but increasingly quadrivalent inactivated vaccines (QIV), can protect both ferrets^56^, ^57^ and humans^58^, ^59^ from infection and disease. Protection is mediated through neutralising antibodies targeting a cluster of epitopes surrounding the viral receptor‐binding domain (RBD) within the highly variable hemagglutinin (HA) head domain (HA1).^60^, ^61^ Inactivated influenza vaccines significantly reduce mortality rates in children^62^, ^63^ and severe disease in adults.^58^, ^64^ However, vaccine protection is notoriously strain‐specific, and mismatches between vaccine and circulating strains through antigenic drift lead to low vaccine efficacy.^65^, ^66^, ^67^

There are global efforts to increase the breadth of protection of influenza vaccines, with an eventual goal of universal protection (reviewed in^68^), and most strategies have been evaluated in ferret models. A non‐exhaustive list of strategies to induce heterosubtypic immunity against influenza evaluated in ferrets include: HA stem vaccination^69^, ^70^, ^71^; prime‐boost with chimeric HA‐based vaccines^72^; use of conserved influenza proteins such as nucleoprotein (NP),^73^, ^74^, ^75^ matrix‐1 (M1),^74^, ^76^ matrix‐2 (M2)^75^, ^77^ and RNA polymerase subunit B1 (PB1)^74^; replication‐deficient viruses^78^, ^79^, ^80^, ^81^; live attenuated formulations^82^, ^83^, ^84^; the use of potent adjuvants such as Protollin,^85^ glucopyranosyl lipid adjuvant—aqueous formulation (GLA‐AF),^86^ CoVaccine HT,^87^ cationic adjuvant formulation^88^ and poly‐g‐glutamic/chitosan nanogel^89^; *Escherichia coli*‐derived vaccines^90^; DNA, mRNA and viral vector vaccines^75^, ^91^, ^92^, ^93^; and the use of virus‐like particles (VLP).^76^ Additional examples of important ferret studies include (but are not limited to) evaluating the influence of changing the route of influenza inoculation on subsequent immunity^94^ and the use of neuraminidase (NA) inhibitors as prophylaxis.^95^

## 4 FERRETS AS AN IMMUNOLOGICAL MODEL FOR STUDYING INFLUENZA

The utility of ferrets for incisive immunological studies is hampered by limited reagents to study ferret immunity and a paucity of background knowledge about the ferret immune system. Some insights into ferrets’ immunological responses to influenza have been gained by indirect measurements of immune gene expression such as quantitative RT‐PCR (qRT‐PCR), transcriptome analysis or oligonucleotide microarrays.^22^, ^96^, ^97^, ^98^, ^99^, ^100^ For example, assessing the differential expression levels of innate and adaptive immune genes in the lungs following primary or secondary 2009 H1N1pdm infection revealed upregulation of interferon‐stimulated genes involved in antiviral responses such as C‐X‐C motif chemokine 10 (CXCL10), 2′‐5′ oligoadenylate synthase 1 (OAS1), interferon regulatory factor 1 (IRF1) and radical S‐adenosyl methionine domain containing 2 (RSAD2) as well as chemokines such as CXCL16 and C‐C motif chemokines 3, 4 and 5 (CCL3, CCL4 and CCL5).^98^ Similarly, the degree of disease severity, virus shedding and transmission in ferrets has been associated with tumour necrosis factor (TNF) and interleukin‐6 (IL‐6) mRNA expression in the upper respiratory tract.^97^ However, mRNA levels can correlate poorly with protein levels,^101^ and techniques such as flow cytometry, bead arrays and immunohistochemistry would facilitate direct measurement of immune marker expression. To date, flow cytometric or microscopy techniques have been limited in ferrets by the lack of suitable antibodies specific for ferret immune cell markers.

Screening for cross‐species reactivity has identified antibody clones recognising ferret T‐cell markers such as CD3 and CD8, and an intracellular B‐cell marker CD79b^102^, ^103^, ^104^ (summarised in Table 1). The utility of such cross‐reactive reagents has been shown experimentally. For example, prime‐boost immunisation using DNA and adenoviral‐based influenza vaccines provided effective protection in experimentally challenged ferrets, with protection correlating to the capacity of CD3+ T cells to express interferon gamma (IFN‐**γ)** following in vitro stimulation on peripheral blood mononuclear cells (PBMCs) with HA peptide pools.^92^ Caution should be taken when using antibodies developed for other species in ferret experiments, as there may be subsets of cells displaying variable reactivity to the antibodies.^105^, ^106^ In addition, currently available anti‐ferret B‐cell antibodies such as CD79α target intracellular epitopes, requiring fixation and permeabilisation. This limits downstream applications such as RT‐PCR or the recovery of antigen‐specific immunoglobulin sequences from sorted B cells. In the absence of cross‐reactive clones, several groups have generated novel monoclonal antibodies specific for ferret cellular markers. For example, novel anti‐ferret CD4‐, CD8‐ and CD5‐specific antibodies were derived by immunising mice with the CD4 ectodomain,^107^ whole CD4 protein^108^ or ferret thymocytes.^83^ Efforts to develop novel antibody‐based reagents to define various ferret immune cell subpopulations are accelerating, particularly through the Centers of Excellence for Influenza Research and Surveillance (CEIRS) network,^109^ and are reviewed in detail further below.

**Table 1:** List of currently available reagents available for studying ferret immune cells

Antigen	Species	Clonality‐Clone	Cell type	Reference(s)
CD20	Human	Polyclonal—RB‐9013‐P	B	108, 163
CD32	Human	Monoclonal—2E1	B	102
CD79a	Human	Monoclonal—HM47/HM57	B	108, 163
CD79b	Human	Monoclonal—ZL9‐2	B	102
IgA	Ferret	Polyclonal—NBP‐72747	B	124
IgA	Canine	Polyclonal	B	102
IgA/G/M	Ferret	Polyclonal	B	108
IgG	Ferret	Polyclonal	B	commercially avaliable
IgG	Mink	Polyclonal	B	102, 175
IgM	Ferret	Polyclonal	B	commercially avaliable
IgM	Human	Polyclonal	B	102
Kappa	Ferret	Monoclonal—multiple	B	118
Lambda	Ferret	Monoclonal—multiple	B	118
Immunoglobulin Heavy chain	Ferret	Monoclonal—multiple	B	118
CD11b	Mouse	Monoclonal—M1/70	Innate	103, 108
CD14	Human	Monoclonal—Tuk40	Innate	102
CD172a	Human	Monoclonal—DH59B	Innate	102
CD163	Swine	Monoclonal—2A10/11	Innate	163
MAC387	Human	Monoclonal—M0747	Innate	163
CD88	Human	Monoclonal—S5/1	Innate	102
SWC3	Swine	Monoclonal—BA1C11	Innate	163
CD43	Mouse	Monoclonal—S7	Pan‐leucocyte	103
LFA‐1	Mouse	Monoclonal—2D7	Pan‐leucocyte	103
Ly6C	Mouse	Monoclonal—AL‐21	Pan‐leucocyte	108
TNF	Mouse	Monoclonal—MP6‐XT22	Pan‐leucocyte	103
MHC‐II	Human	L243	T	108
CD3	Human	Polyclonal—IS503	T	163
CD103	Mouse	Monoclonal—M290	T	103
HLA‐DR	Human	Monoclonal—TAL.1B5	T	163
CD25	Human	Monoclonal—B1.49.9	T	102
CD4	Ferret	CL3.1.5	T	108
CD4	Ferret	Monoclonal	T	107
CD8	Human	Monoclonal—OKT8	T	102, 103, 108
CD8	Ferret	Polyclonal—60001RPO2	T/NK	163
IL‐4	Bovine	Monoclonal—CC303	T	103
Thy1.1	Mouse	Monoclonal—OX‐7	T	103
IFN‐γ	Bovine	Monoclonal—CC302; XMG1.2	T/NK	103

Using existing reagents, innate and adaptive immune responses to influenza infection^52^, ^108^, ^110^ and immunisation^83^, ^111^ have been studied and provide important insights into ferret antiviral immunity. Following influenza challenge of naïve or vaccinated ferrets, there is a transient increase in CD11b+ expressing PBMCs at 2 d.p.i, which decreased to baseline levels by 3 d.p.i, suggesting a role for monocytes/macrophages/neutrophils or antigen‐presenting cells (APC) during early infection.^110^, ^111^ In addition, an infiltration of CD11b+ and MHC‐II+ cells into lungs between 2 and 5 d.p.i was observed, which could represent lung APCs.^110^ CD11b+ cells also increase in secondary lymphoid organs 10 d.p.i,^108^ suggesting a coordinated immune response involving both innate and adaptive responses to resolve infection.

Ferrets have also been employed to examine the spatiotemporal dynamics of B‐ and T‐cell responses after vaccination or infection with different influenza strains.^52^, ^110^ Immunologically naïve or primed ferrets challenged^110^, ^111^ with influenza A virus (IAV) are observed to have transient blood CD4+ T/CD8+ T‐cell lymphopenia.^110^ Correspondingly, influenza‐specific serum IFN‐**γ** responses increased by 5‐7 d.p.i which remain elevated up to 14‐34 d.p.i.^52^, ^83^ This is consistent with the increase in CD8+ frequencies by 10‐34 d.p.i in vaccinated/infected ferrets 83, 108 and influenza‐reactive CD4+ cells by 8‐34 d.p.i in draining lymph nodes.^52^, ^83^ Further comparison of IFN‐**γ**‐expressing T‐cell responses during an infection in lung and blood PBMCs suggested H1 and H3 establish infection in different organ compartments, since IFN‐**γ**‐expressing T‐cell responses in the lungs of H3‐infected ferrets were significantly lower than H1‐infected ferrets.^52^ This is consistent with other studies showing limited replication of H3 viruses in ferret lungs.^111^, ^112^, ^113^

The influenza protein specificity of the T‐cell response to influenza is beginning to be explored in ferrets using IFN‐**γ** expression assays and other standardised protocols.^108^, ^114^ DiPiazza et al^108^ found that the majority of bulk memory CD4+ T‐cell responses were specific for the M1 protein whereas non‐structural protein (NS) was mainly the target for CD8+ T‐cell responses, and hierarchal responses were found to change over time without preferential retention of immunodominant specificities.^115^ In comparison, cross‐reactive ferret CD4+ T cells recognise HA and NA epitopes preferentially whereas CD8+ T cells mount immune responses towards M1, NS2 and RNA polymerase subunit A (PA), with NP as a significant antigenic target.^104^ Interrogation of ferret T‐cell responses with improved reagents will increase our understanding of immunodominance hierarchies analogous to studies performed in other animal models and humans (reviewed in^116^).

Markers targeting B‐cell antigens such as CD79a, CD20 and surface immunoglobulin also allow ferret B cells to be examined by immunohistochemistry and flow cytometry in ferret tissues.^108^, ^117^, ^118^ B‐cell frequencies are also transiently decreased 2 d.p.i^110^, ^111^ after infection, with a corresponding increase in secondary lymphoid organs 2‐5 d.p.i.^110^ The number of major histocompatibility complex (MHC‐II) expressing cells also increased, with no significant difference in surface immunoglobulin‐positive cells by 10 d.p.i.^108^ Influenza‐specific ASCs were also increased by 37 d.p.i, highlighting the role of B cells in the resolution of infection.^83^ These observations suggest changes in the maturation status of B cells after activation and mirror observations in humans and mice (as reviewed in^119^). MHC‐II is upregulated in B cells to induce germinal centre formation (GC) through cognate interactions with T follicular helper cells (T_FH_). This has been demonstrated in mice, where the ablation of MHC‐II expression in mice led to a decrease in influenza‐specific IgG and IgA titres and decreased survival rates.^120^ Surface immunoglobulin expression in antibody‐secreting plasma cells (ASCs) is also downregulated, consistent with the decrease in the number of surface immunoglobulin expressing HA‐reactive GC cells by 14 d.p.i.^121^

Similar to T cells, B‐cell HA immunodominance hierarchies have been studied widely.^122^ A key target for broader antibody responses is the conserved stem domain (HA2) of HA; however, immunodominant responses against HA1 often limit antibody responses to variable regions, leading to escape from host responses.^123^ Different vaccination strategies to induce broadly protective antibody responses have been studied in ferrets.^124^ Using purified ferret immunoglobulins and cross‐reactive polyclonal immunoglobulin antisera from mink, goat, canine and rabbits, a ferret immunoglobulin class‐specific ELISA was developed.^102^ By exposing ferrets to recombinant HA constructs with exotic HA head domains via infections and vaccinations (H9/H8/H5 head domain with H1 stem domain), immunologically subdominant anti‐HA stem responses were induced as measured by ELISA.^124^ Polyclonal stem‐reactive antibodies were detected serologically and protected ferrets against pH1N1 challenge in the presence of low neutralisation activities as measured by microneutralisation assays. This is consistent with studies in other mammalian models and humans suggesting that Fc‐mediated functions are important for HA2‐mediated protection (as reviewed in^125^). Further delineation of such immunodominance hierarchies in HA at the monoclonal antibody level and functional characterisation of HA2‐specific antibodies are reviewed in detail further below.

The effects of prior infection on host susceptibility to re‐infection, that is viral interference, have also been studied in ferrets.^126^ Ferrets sequentially challenged with B/Victoria and B/Yamagata viruses display decreased virus shedding, which correlated with the induction of high frequencies of cross‐reactive IFN‐**γ**‐expressing T‐cell responses between initial infection and heterologous challenge.^127^ Infection with A(H1N1)pdm09 was also shown to prevent HRSV infections in ferrets, though no IFN‐**γ** responses or cross‐reactive serological responses were observed, suggesting different underlying mechanisms driving viral interference between unrelated viruses.^128^This observation mirrors epidemiological studies in humans, where peak incidences of HRSV infections in humans were delayed by influenza A outbreaks (reviewed in^128^).

Several key questions remain unanswered in ferrets with regard to influenza‐specific immune responses. First, chemotactic signals important for the spatiotemporal distribution of immune cells are still largely unknown in ferrets; for example, influenza‐infected epithelial cells secrete CCL‐2 which recruits monocytes to the lungs during early infection and may be associated with acute lung injury^129^ during severe infections. Secondly, different innate immune subsets such as natural killer (NK) cells, dendritic cells (DCs), monocyte/macrophages and granulocytes have also yet to be studied in detail, and development of reagents to allow the delineation of these cell populations (reviewed in^130^, ^131^, ^132^, ^133^) will enable a more detailed picture of early immune responses in ferrets. Thirdly, while adaptive immune responses have been studied in ferrets, markers to delineate B/T‐cell subpopulations will be useful to study long‐term protection against influenza infection (as reviewed in^134^). Examples include CD62L and CD44 for naïve and memory T cells, and IgD and CD27 for naïve and memory B cells, respectively.

## 5 FERRETS AS AN IMMUNOLOGICAL MODEL FOR OTHER EMERGING VIRAL DISEASES

In addition to influenza, the ferret serves as a critical model for other important human pathogens such as SARS‐CoV, pneumoviruses (HRSV and HMPV), Ebola virus and henipaviruses. While these infections remain less characterised in comparison with influenza, the ferret provides a platform to examine disease pathogenesis and transmission and to evaluate potential vaccine efficacy. However, like influenza, evaluation of host immune responses in ferrets is commonly restricted to gene expression analyses.

### 5.1 SARS‐CoV

SARS‐CoV infection causes acute respiratory distress in humans with mortality rates of up to 10%.^135^ While worldwide outbreaks have not been reported since 2004, there is still a lack of vaccines and effective treatment measures. Ferrets display clinical signs of infection such as elevated body temperatures, sneezing, increase in lymphocyte counts and lesions in the respiratory tract and alveolar oedema^7^ and are therefore a good mammalian model to study the pathogenesis of SARS‐CoV (reviewed in^7^, ^136^) and evaluate vaccines (reviewed in^14^, ^137^, ^138^, ^139^). In terms of immunity, ferrets exhibit strong antiviral interferon responses after infection and vaccination as measured by interferon response gene expression levels.^5^, ^13^ However, leucocyte counts and interferon‐related gene expression were decreased upon re‐infection,^5^ suggesting that innate immune dysregulation is a possible mechanism of pathogenesis, though a protective antibody response was also evident during attempts to re‐infect ferrets.^8^

### 5.2 Henipavirus

Emerging viruses belonging to the *Paramyxoviridae family* (Henipaviruses) can cause severe respiratory illness and/or encephalitis in humans. Ferrets infected with henipaviruses exhibit similar symptoms as humans including respiratory signs such as cough and nasal discharge, neural signs such as depression,^32^ and high mortality rates with most experimentally infected ferrets succumbing within 1 week.^31^ While the virus is detected in pharyngeal and rectal secretions, it is currently unclear if ferrets could serve as a transmission model for the disease.^31^, ^32^ Ferrets infected intranasally with henipaviruses similarly display clinical illness.^31^, ^34^ Assessment of immune gene expression by Leon et al^31^ in both lungs and brain tissues of the infected ferrets revealed upregulation of macrophage markers such as CD40 and CD80 in both lung and brain tissues, whereas lymphocytic markers were unchanged in the lungs.

### 5.3 Respiratory syncytial virus and metapneumovirus

RSV and HMPV cause severe respiratory disease in young children, the elderly and immunocompromised patients. Both RSV and HMPV readily infect ferrets but in general do not exhibit signs of disease.^15^, ^20^, ^21^ Nevertheless, ferrets have proven to be a useful model to study RSV. Several groups have successfully infected ferrets with a wild‐type strain of human RSV and demonstrated efficient replication in both the upper and lower respiratory tracts of adult ferrets,^15^, ^20^ consistent with humans where infection is often limited to the upper respiratory tract.^140^ Immunocompromised ferrets, induced by oral administration of immunosuppressive drug mycophenolate mofetil (MMF), demonstrate prolonged RSV shedding and effective contact transmission to both immunocompetent and immunocompromised ferrets,^18^ confirming antiviral immunity in the ferret can curtail viral replication. An assessment of lung immune gene expression in ferrets infected with RSV demonstrated an upregulation of proinflammatory cytokines such as interleukin‐1 alpha (IL‐1α) and interleukin‐1 beta (IL‐1β) by 5 d.p.i which coincided with maximum levels of RSV mRNA, while levels of other cytokines such as interferon alpha (IFN‐α) and IFN‐**γ** remained unchanged.^20^ In terms of humoral responses, increased serum titres of fusion (F) glycoprotein antibodies were seen by 15 d.p.i^20^ that were protective against re‐infection.

### 5.4 Ebola virus

Ebola virus disease (EVD) is caused by a zoonotic virus from the *Filoviridae* family of viruses.^28^ This disease can transmit from human to human and causes acute and often fatal disease. Ferrets are able to be directly infected with the Zaire, Bundibugyo and Sudan Ebola strains,^22^, ^23^ which have previously caused major human outbreaks. Ferrets display hallmarks of pathological processes of human lethal infections such as petechial rashes, reticulated pallor of the liver and splenomegaly.^23^, ^24^ Transmission has also been reported in ferrets.^141^ As for immunological studies, transcriptomic sequencing in ferrets infected with lethal doses (1000 plaque‐forming units (PFU)) of the Makona variant of *Zaire ebolavirus* revealed upregulation of proinflammatory‐related genes such as interferon activation, Toll‐like receptor signalling, interleukin‐1/6 responses and coagulation cascades by 5 d.p.i.^142^

## 6 KEY KNOWLEDGE GAPS TO ADDRESS IN ORDER TO IMPROVE THE IMMUNOLOGICAL UTILITY OF FERRET MODELS

While the ferret model has unique potential for informative studies into pathogenic viral infections as noted above, addressing several key knowledge gaps will substantially advance the ferret as an immunological model.

### 6.1 Immunogenetics

There is a lack of well‐annotated, ferret genomic sequence information to characterise immune responses, limiting the scope of molecular analyses that can be performed; ferret T/B‐cell receptor repertoire analysis is currently not possible. Next‐generation sequencing (NGS) has become increasingly important for immunological research and has led to the generation of huge amounts of data and the development of tools for data extraction and analysis. An important aspect of T‐ and B‐cell research is the immune cell receptor repertoire during an infection and the effects of allelic variation of important immunological molecules such as major histocompatibility complex (MHC) on host immune responses. A draft copy of the ferret genome is available,^62^ but genes coding for B‐ or T‐cell receptors have yet to be fully annotated and validated. Genomic sequencing and assembly of closely related species such as minks^143^ are also far from complete, though several similarities such as genome size and relative abundance of repeat elements have been found. In comparison, high‐quality draft genome assemblies for dogs and cats are available and have been used for genome‐wide association studies^144^ and identification of single nucleotide polymorphisms (SNPs).^145^ The identification of SNPs in immunoglobulin genes is useful for distinguishing between somatically mutated B‐cell receptor sequences and germline variants in affinity‐matured antigen‐specific B‐cell populations. There are currently databases of immunoglobulin sequences for well‐established animal models such as those found in the international ImMunoGeneTics (IMGT) information system database^146^ and have been useful for identifying somatic hypermutations in immunoglobulin sequences.^147^ A curated and annotated database of immune gene sequences is a prerequisite for PCR primer design and post‐sequencing data analysis used to recover and express antibodies from single‐sorted B cells^148^ and recombinant T‐cell receptors.^149^

### 6.2 Future T cell–specific reagents for ferrets

Future development of markers to delineate more T‐cell subsets will increase the utility of the ferret as an immunological model; a recent report listed several important ferret T cell–specific antibodies to be in production at the CEIRS such as CD4, CCR7, CD3e, CD40, CD40L, CD44, CD62L, CD69, CD103, PD‐1, CXCR3, CXCR5, IL‐7R and IL‐15R.^109^

### 6.3 Future B cell–specific reagents for ferrets

To increase our understanding of antibody responses in ferrets, flow cytometric reagents that are able to delineate B‐cell subsets are required. Important ferret B cell–specific antibodies that are in production at the CEIRS include CD83, CD86, CD95, CD19, CD20, CD25, CD27, CD38, CD138 and FcR.^109^

### 6.4 Current and future markers for ferret myeloid lineage cells

Several markers defining innate cell populations in mice and humans such as CD11b^103^, ^108^ and CD14^102^ have also been reported to cross‐react with ferret leucocytes and have been utilised to characterise ferret innate immune responses. However, these markers have also been found to be expressed in non‐myeloid lineages in humans,^150^, ^151^ and other markers such as CD16^152^ and CD66^153^ will be required to better define myeloid cell populations.

### 6.5 Ferret immunoglobulin subclass and Fc receptors

Ferret immunoglobulin subclass and Fc receptors are not well studied. Currently, only one IgG subclass that has been identified in ferrets, while four different IgG subclasses have been identified in other carnivores such as dogs^154^ and minks^155^ and three in felines,^156^ suggesting other unidentified ferret IgG subclasses may exist. The diversity and function of ferret Fc receptors are also unknown. Different IgG and Fc receptor subclasses in humans and mice have shown to be important for different antibody‐mediated effector functions such as antibody‐dependent cellular cytotoxicity (ADCC). Human Fc gamma receptor IIIa (FcγRIIIa; FcγRIV in mice) is the main receptor involved in ADCC, and human IgG3 (mouse IgG2a) followed closely by human IgG1 (mouse IgG2b) displays the highest affinities for this receptor. Advanced assays for ADCC and other Fc‐mediated responses to influenza have been developed in recent years in both NHP^157^ and humans.^158^, ^159^ Such non‐neutralising mechanisms have been shown to be important for broadly protective responses against antigenically distinct strains of influenza^157^, ^160^, ^161^ and development of universal influenza vaccines. Many of the current ADCC and related assays rely on the detection of Ab‐mediated activation of NK cells to express cytokines such as IFN‐γ or degranulation markers such as CD107a,^162^ but there are currently no reagents to differentiate NK cells from other cytotoxic lymphocyte populations such as CD8+ T cells.^163^ While a ferret‐specific T‐cell IFN‐γ expression assays^164^ to measure CD8 T‐lymphocyte activation have been developed and validated, the future elucidation of corresponding antibody/Fc receptor ferret orthologues will enable ferrets to be used to evaluate ADCC responses.

### 6.6 Antigenic recognition of major influenza proteins

Epitope mapping studies using either human or murine monoclonal antibodies have greatly increased our understanding of influenza viral evolution and allowed the identification of major HA epitopes and pathways of immune escape. While factors that determine the dominant escape mutants are still unknown, such studies have the potential to improve the process of influenza vaccine design. The inability to isolate monoclonal antibodies from ferret B cells has limited studies into the antigenic recognition of influenza proteins in ferrets. Confirming epitope‐specific recognition of HA at the monoclonal antibody level in ferrets is critical, as there have been several reports that human and ferret serum antibodies can display variable antigenic recognition.^165^, ^166^ This is critically important as antisera from infected ferrets is widely used in HI assays as part of the strain determination process for influenza vaccines.^167^, ^168^

Major antigenic sites of HA have been localised by generating viral escape mutants in the presence of influenza‐specific murine^169^, ^170^ or human^171^ monoclonal antibodies*,* which for H1N1 viruses have been termed Sa, Sb, Ca1, Ca2 and Cb.^170^ A study of influenza A (IAV) HA antigenic sites using engineered viruses with mutations in each of the antigenic sites surrounding the RBD revealed species‐specific differences in antibody recognition.^166^ HI activities against H1N1 (A/Michigan/45/2015) mutants as described revealed differences in antigenic epitopes recognised by mice, guinea pigs, ferrets and adult humans using serum samples. For example, neutralising antibodies in adult humans recognised mostly Sb and Sa, while responses in ferrets were mostly directed to Sa.^166^ Another similar study characterising humoral responses against influenza B (IBV) using reverse‐engineered viral mutants showed that the proportion of human antibodies targeting non‐canonical antigenic sites of IBV HA are comparable to canonical antigenic sites (120 loop, 150 loop, 160 loop and 190 helix).^172^ This is in contrast to IBV‐specific responses in mice and ferrets, where most serum antibody is directed against canonical antigenic sites only. These results suggest inter‐species differences in humoral recognition of the influenza HA. Other studies lend support, with a head‐to‐head comparison between humans and ferrets immunised or infected with various seasonal H3 vaccine strains showing key differences in serological recognition of viral HA^173^ based on molecular modelling and antigenic cartography. Similarly, cross‐reactive H1N1 antibody responses in ferrets are not predictive of cross‐reactive responses in humans,^174^ confirming fundamental differences in B‐cell responses between humans and ferrets.

## 7 CONCLUSION

Ferrets have tremendous utility for studying the pathogenesis and transmission of several human respiratory diseases and for pre‐clinical evaluation of vaccines. Ferrets are a critically important model that directly impacts human seasonal influenza vaccine selection and the pre‐clinical development of vaccines for other emerging diseases. However, knowledge gaps limit the in‐depth assessment of any immune mechanisms that may underpin transmission, protection and/immunopathology of these viral diseases in the ferret model. There is an urgent need for novel reagents with well‐validated and specific targets to resolve different immune cell populations. Improving the ferret model to enable the application of insightful modern immunological tools, such as single‐cell B‐cell receptor sequencing, next‐generation sequencing platforms and other bioinformatics tools, will greatly enhance the informative value of the ferret model which will in turn lead to better immunological interventions for human respiratory diseases.

## CONFLICT OF INTEREST

The authors declare that the research was conducted in the absence of any commercial or financial relationships that could be construed as a potential conflict of interest.

## AUTHOR CONTRIBUTIONS

JW performed literature review and prepared the manuscript. DL, SK and AW edited and provided comments. All authors contributed to the formulation of this scientific research topic.
